# A Four-Year Trend of Ceftriaxone Resistance and Associated Risk Factors Among Different Clinical Samples in Wad Medani, Sudan: A Cross-Sectional Retrospective Study

**DOI:** 10.7759/cureus.64184

**Published:** 2024-07-09

**Authors:** Yousif B Hamadalneel, Marwa F Alamin, Alaaeldeen M Attaalla

**Affiliations:** 1 Clinical Pharmacy, Faculty of Pharmacy, University of Gezira, Wad Medani, SDN; 2 Molecular Biology, Institute of Endemic Diseases, University of Khartoum, Khartoum, SDN; 3 Internal Medicine, Faculty of Dentistry, University of Gezira, Wad Medani, SDN

**Keywords:** trend, ceftriaxone, resistance, patterns, wad medani, sudan

## Abstract

Introduction

In sub-Saharan Africa, including Sudan, there is commonly no local data on the bacterial profile or antibiotic resistance pattern. Therefore, to bridge these gaps, this study aimed to evaluate ceftriaxone resistance patterns and associated risk factors among different clinical samples.

Methods

This study was a laboratory-based, retrospective, cross-sectional study. All clinical specimens were obtained from patients at Wad Medani and examined at the Pathology Center for Diagnosis and Research, Faculty of Medicine, University of Gezira, Sudan, from January 2020 to October 2023.

Results

Overall, 1784 specimens exhibited bacterial growth over four years. Of these, 1260 (70.6%) were females. Approximately one-third of the 588 (33%) studied patients were aged 30 to 44 years. Of the studied samples, 1108 (62.1%) were urine, and 465 (26.1%) were wound swabs. *Staphylococcus aureus* (697, 39.1%) and *Escherichia coli* (656, 36.8%) were the most frequently encountered bacteria. Generally, ceftriaxone resistance has been evaluated in 150 positive culture samples. The overall ceftriaxone resistance rate was 106 (70.7%). The greatest proportion of ceftriaxone resistance was observed in 4/4 (100%) of *Klebsiella* spp. and 66/82 (80.5%) of *E. coli* strains. The type of isolate (95% Cl, *p-value*; 0.006) and type of bacterial stain (95% Cl, *p-value* 0.013) have been significantly associated with ceftriaxone resistance, in which Gram-negative bacteria had a greater resistance rate of 98/132 (74.2%) than Gram-positive bacteria 8/18 (44.4%).

Conclusions

This study revealed a high rate of ceftriaxone resistance. The most resistant bacteria were *Klebsiella* spp. and *E. coli*. The type of isolate and bacterial stain were significantly associated with ceftriaxone resistance. Therefore, hospitals should immediately and significantly modify their antibiotic prescription policy to give doctors a consistent strategy for the rational, safe, and effective administration of antibiotics.

## Introduction

Antimicrobial resistance has become a pressing global health concern and is one of the top 10 threats to global health [1]. The emergence of antimicrobial resistance as a consequence of the use, abuse, and overuse of antibiotics restricts the ability of these medications to treat patients [2].

Antimicrobials are crucial for lowering the global burden of infectious and communicable diseases [3]. Among the various antibiotics, ceftriaxone, a third-generation cephalosporin, remains an essential therapeutic option for a variety of bacterial infections. Its great efficacy, low risk of toxicity, broad coverage, and clinical versatility have made it a cornerstone in the management of conditions, including urinary tract infections, skin and soft tissue infections, pneumonia, bone infections, and abdominal infections [4].

Many studies worldwide reported that the prevalence of ceftriaxone resistance has been steadily rising [5,6]. Two studies conducted in Gamby and Jimma Teaching Hospitals, Ethiopia, reported that ceftriaxone exhibited overall resistance rates of 57.2% and 56.5%, respectively [5,6]. Furthermore, these studies revealed that the most common ceftriaxone-resistant bacteria were *Escherichia coli *(*E. coli*) and *Staphylococcus aureus* (*S. aureus*) [5,6]. In Sudan, no direct studies have been conducted to address this problem. A study carried out to investigate whether ceftriaxone was used appropriately in the internal medicine wards of the Wad Medani Teaching Hospital, Sudan, revealed that 91.1% of the patients had empirical indications for ceftriaxone, the majority had an incorrect duration in 51.1%, in addition to an inappropriate dose and a frequency of 59% and 68.9%, respectively [7].

In developing countries, particularly in sub-Saharan Africa, including Sudan, the current treatment guidelines focus on the empiric treatment of infections such as ceftriaxone, while a World Health Organization (WHO) global report on the surveillance of antimicrobial resistance revealed that there is commonly no local data on antibiotic resistance and an absence of data on the most prevalent pathogens. However, this approach may worsen patient outcomes and increase bacterial resistance [8,9]. In Sudan, there are no local policies on antimicrobial resistance. Furthermore, there are no established protocols or guidelines concerning the use of antimicrobial agents in research settings; instead, medical professionals rely on their expertise and familiarity with these agents. Furthermore, according to a recent report on antimicrobial resistance in the WHO African Region, most of the studies were from Ethiopia, and there is an absence of data on antimicrobial resistance in Sudan [10]. In Wad Medani, Sudan, there is no published data on the ceftriaxone resistance profile. Therefore, to bridge these gaps, this study aimed to evaluate ceftriaxone resistance patterns and associated risk factors among different clinical samples from Wad Medani, Sudan.

## Materials and methods

Study setting and design

This study was conducted in Wad Medani, Gezira State, Sudan, and the research utilized a retrospective, cross-sectional study design.

Sample size and data collection

All clinical specimens were obtained from patients at Wad Medani and examined at the Pathology Center for Diagnosis and Research (PCDR), Faculty of Medicine, University of Gezira, Sudan, from January 2020 to October 2023.

Inclusion and exclusion criteria

All samples tested at the PCDR for culture and drug sensitivity were included, except for bacteria not recommended for ceftriaxone testing according to the Clinical and Laboratory Standard Institute (CLSI) guidelines [11].

Sample collection

Different clinical samples (urine, blood, cerebrospinal fluid (CSF), swabs (wound, ear, and vaginal), pus, and body fluid (pleural and ascitic)) were collected from the study population by applying standard microbiological approaches; for example, 2 mL, 5 mL, and 10 mL of venous whole blood were aseptically collected from neonatal, pediatric, and adult blood, respectively [12]. All specimens were aseptically sent to the microbiology laboratory of the PCDR for culture and drug sensitivity tests.

Study variables

Sex, age, year, type of bacterial stain, type of isolate, and type of clinical sample were considered independent variables, while ceftriaxone resistance was considered the dependent variable in this study.

Definition of study variable

Coliform: Includes bacteria from families other than *Klebsiella *and *E. coli*, such as *Serratia*, *Enterobacter*, *Citrobacter*, and others.

Identification of the isolated organism

The collected specimens were inoculated onto MacConkey agar and blood agar plates for urine samples using a calibrated loop (0.001 mL) [12]. Cultures were incubated in an aerobic atmosphere at 37°C for 24 hours. A colony count of ≥10^5^ colony-forming units per millilitre (CFU/mL) for midstream urine was identified as a positive urine culture [13]. Similarly, all plates were incubated accordingly based on their specimen type and the organism expected [12].

Gram staining, confirmatory biochemical testing, and morphological characteristics were utilized to identify the bacterial isolates for all positive cultures. To identify Gram-negative bacteria, inoculation on MacConkey agar plates was used. Next, biochemical tests including urease and oxidase tests, hydrogen sulfide (H2S) production, indole formation, and utilization of citrate/carbohydrates were performed. Additionally, the catalase reaction, coagulase test, optochin test, bacitracin test, and hemolytic activity test on blood agar were used to identify Gram-positive bacteria [12].

Antimicrobial susceptibility

In accordance with CLSI 2020 standards, the susceptibility of the bacterial isolates to ceftriaxone was determined on Mueller-Hinton agar plates (Oxoid, England) using the Kirby-Bauer disk diffusion method [11]. To interpret zone diameters, the CLSI 2020 guideline breakpoints were utilized [11].

Quality control

Throughout the entire laboratory work process, methods for quality assurance were used as normal procedures to ensure the validity of the results. Before use, the staining reagents, antibiotic discs, and culture media were examined for normal shelf life [14]. Following preparation and autoclaving at 121°C for 15 minutes, every culture plate and antibiotic disc was kept refrigerated at the specified temperature. The standard reference bacterial strains were investigated as a positive control on agar plates with biochemical assays and antibiotic discs [14]. The samples were processed carefully by highly qualified microbiologists.

Statistical analysis

The data was analyzed using the Statistical Package for the Social Sciences (IBM SPSS Statistics for Windows, IBM Corp., Version 27.0, Armonk, NY). Frequencies (percentages) were used to present qualitative data. The chi-square test and Fisher’s exact test were used to determine factors significantly associated with ceftriaxone resistance after checking the applicability conditions. A confidence interval (CI) of 95% and a *p-value* less than 0.05 were considered to indicate statistical significance.

Ethical approval

The study protocol was approved by the Ethical Committee, Ministry of Health, Gezira State, Sudan (12/6/2023). Patient consent was also waived by the Ethical Committee, Ministry of Health, Gezira State, Sudan (12/6/2023), because this was a retrospective study in which the samples were collected for diagnostic purposes independently of the study, and the data were provided to us anonymously.

## Results

Sociodemographic characteristics

A total of 1784 samples exhibited bacterial growth over four years. Of these, 1260 (70.6%) were females. Approximately one-third of the 588 (33%) studied patients were aged 30 to 44 years (Table 1). Most of the studied samples 792 (44.4%) were isolated in 2022. Of the studied samples, 1108 (62.1%) were urine, 465 (26.1%) were wound, and 86 (4.8%) were blood (Table 2).

**Table 1:** Sociodemographic characteristics of study participants in Wad Medani

Characteristics	Number (%)
Sex	
Male	524 (29.4)
Female	1260 (70.6)
Age group in year	
0-14	128 (7.2)
15-29	254 (14.2)
30-44	588 (33)
45-59	379 (21.2)
≥ 60	435 (24.4)
Years	
2020	180 (10.1)
2021	286 (16)
2022	792 (44.4)
2023	526 (29.5)

**Table 2:** Clinical samples of study participants in Wad Medani CSF: cerebrospinal fluid

Sample	Number (%)
Urine	1108 (62.1)
Blood	86 (4.8)
CSF	17 (1)
Wound swabs	465 (26.1)
Vaginal swabs	77 (4.3)
Body fluid	7 (0.4)
Pus	9 (0.5)
Ear swabs	15 (0.8)
Total	1784 (100.0)

Bacterial profile

**Figure 1:**
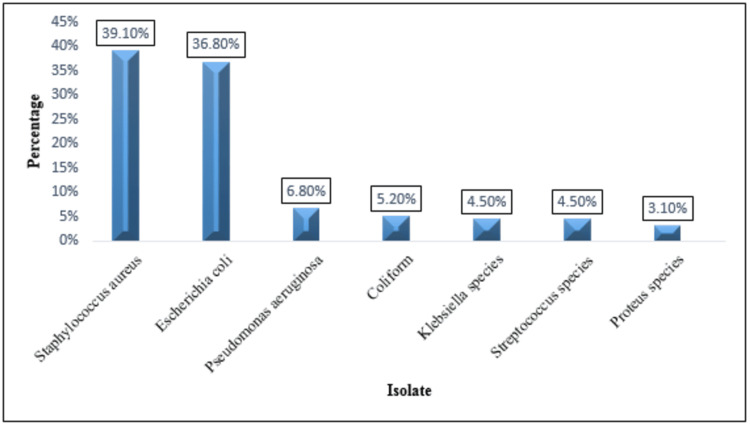
Isolated bacteria from different clinical samples Coliform: bacteria from family other than *Klebsiella *and *E. coli *(*Serratia, Enterobacter, Citrobacter, *etc*.*).

Seven types of bacteria were isolated. Of these, *S. aureus* (697, 39.1%) and *E. coli* (656, 36.8%) were the most frequently encountered bacteria, while the least common bacteria isolated were *Proteus *species (spp) (56, 3.1%). However, 777 (43.6%) of the isolates were Gram-positive, while the majority (1007, 56.4%) were Gram-negative bacteria (Figure 1).

Ceftriaxone resistance pattern

In total, 150 positive culture samples were tested for ceftriaxone resistance. The overall ceftriaxone resistance rate in the isolated bacteria was 70.7% (106/150). Most of the bacteria had resistance levels higher than 50%. The highest percentage of ceftriaxone resistance rate was noted in *Klebsiella *spp. (4, 100%) and *E. coli* (66, 80.5%), while the lowest bacterial resistance rate was observed for *Streptococcus* spp. (8, 44.4%) (Table 3).

**Table 3:** Factors associated with ceftriaxone resistance in Wad Medani (n = 150) ^▲^ Pearson’s Chi-squared and Fisher's exact test * A significant *p-value* at a confidence interval of 95% - Not applicable ^╬^Bacteria from family other than *Klebsiella *and *E. coli *(*Serratia*, *Enterobacter*, *Citrobacter*, etc.). N (%): number (percentage)

Characteristics	N (%)	*p-*value^▲^
Sex		0.152
Male	58/76 (76.3)	-
Female	48/74 (64.9)	-
Year		0.157
2020	54/71 (76.1)	-
2021	7/11 (63.6)	-
2022	12/23 (52.2)	-
2023	33/45 (73.3)	-
Age group in year		0.310
0-14	3/7 (42.9)	-
15-29	21/32 (65.6)	-
30-44	28/39 (71.8)	-
45-59	22/32 (68.8)	-
≥ 60	32/40 (80)	-
Type of clinical sample		0.604
Urine	5/8 (62.2)	-
Cerebrospinal fluid (CSF)	3/5 (60)	-
Pus	5/8 (62.5)	-
Wound swabs	86/117 (73.5)	-
Vaginal swabs	7/12 (58.3)	-
Type of bacterial stain		0.013^*^
Gram-positive	8/18 (44.4)	-
Gram-negative	98/132 (74.2)	-
Type of isolate		0.006*
Escherichia coli	66/82 (80.5)	-
*Klebsiella *species	4/4 (100)	-
*Proteus* species	18/32 (56.3)	-
*Streptococcus* species	8/18 (44.4)	-
*Coliform*^╬^	10/14 (71.4)	-

Factors associated with ceftriaxone resistance

**Figure 2:**
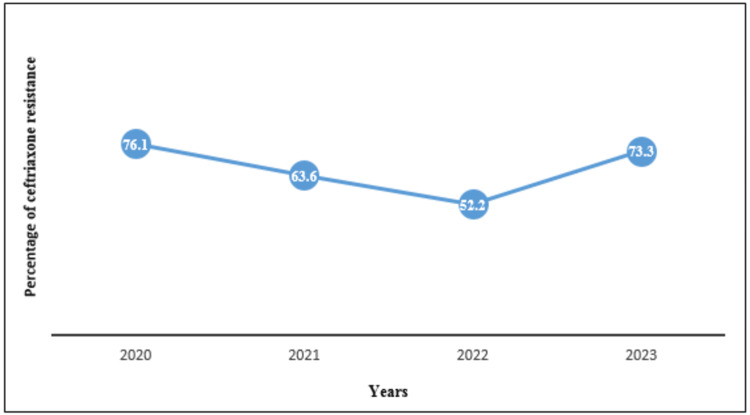
A four-year trend line of the ceftriaxone resistance rate

The ceftriaxone resistance rate was examined against the patient's age, sex, type of isolate, type of bacterial strain, and type of clinical sample. There was a statistically significant association between ceftriaxone resistance and the type of isolate (95% Cl, *p-value* 0.006), in which *Klebsiella* spp. 4/4 (100%) and* E. coli* 66/82 (80.5%) were the most resistant to ceftriaxone (Table 3). In addition, there was a statistically significant association between ceftriaxone resistance and the type of bacterial stain (95% Cl, *p-value* 0.013), in which Gram-negative bacteria had a greater resistance rate 98/132 (74.2%) than Gram-positive bacteria 8/18 (44.4%) (Table 3). A line graph of ceftriaxone resistance showed that ceftriaxone resistance decreased gradually during 2020, 2021, and 2022, going from 54/71 (76.1%) to 7/11 (63.6%) to 12/23 (52.2%). However, ceftriaxone resistance spiked to 33/45 (73.3%) in 2023 (Figure 2).

## Discussion

Globally, antimicrobial resistance is increasing and spreading [1]. This is a challenge for caregivers and medical professionals worldwide. This cross-sectional retrospective study reported that the overall rate of ceftriaxone resistance among different isolated bacteria was 70.7%. There are great variations in ceftriaxone resistance among different studies. This could be due to variations in the study population, region, development, pregnancy status, or genetics. These findings are greater than those of two studies carried out at Gamby and Jimma Hospital, Ethiopia, which showed that the overall ceftriaxone resistance rates were 57.2% and 56.5%, respectively [5,6]. Furthermore, our findings were greater than those of a study conducted in Libya among different clinical specimens, which reported that the total resistance to ceftriaxone was 48.8% [15]. The high resistance rate to ceftriaxone in this study could be linked to malfunctioning activity related to drug use, and this was supported by a study conducted in Wad Medani, Sudan, to evaluate ceftriaxone use; the authors stated that ceftriaxone was given inappropriately regarding the frequency and duration, 68.9% and 51.1%, respectively [7].

The most common isolated microorganism in this study was *S. aureus*, followed by *E. coli*. This finding was similar to that of a study conducted in Pakistan, which revealed that *S. aureus *was the most predominant isolate (30%) followed by *E. coli* (25%) [16]. The prevalence of *E. coli *in this study was similar to that reported in a study performed at Gondar Hospital, Ethiopia, in which the prevalence was 36% [17], and higher than that reported in two studies conducted in Zambia and Ain Shams University Hospitals, Egypt, in which the prevalence of *E. coli *was 13% and 13.4%, respectively [18,19]. The least common bacteria isolated in this study were *Proteus* spp. (3.1%), which is in line with the findings of two previous studies carried out at Jimma Hospital and Mofid Children’s Hospital, Tehran, which reported that *Proteus* spp. were the least predominant isolates 2.0%, and 0.8%, respectively [6,20]. Additionally, the majority of isolates in our study were Gram-negative (56.4%), which was almost identical to the results of a study conducted at Gamby Hospital, Ethiopia, in which Gram-negative bacteria accounted for 60% of the total isolates [5].

With regard to factors associated with ceftriaxone resistance, there was a statistically significant association with isolated bacteria. The greatest proportion of ceftriaxone-resistant strains was related to *Klebsiella* spp. These findings were supported by a study conducted at Gamby Hospital, which revealed that *Klebsiella *spp. exhibited the highest resistance rate to ceftriaxone (85.7%) with no statistically significant difference [5]. Furthermore, *E. coli *showed 80.5% resistance to ceftriaxone, which is greater than that reported by two studies conducted in Jimma Hospital, Ethiopia, and North India, which reported that *E. coli* exhibited 73% and 71.4% resistance rates to ceftriaxone, respectively [6,21]. In addition, this finding is approximately two times greater than that of a study conducted at Gamby Hospital, Ethiopia, in which *E. coli* had a 47.4% resistance rate to ceftriaxone [5]. Moreover, the rate of ceftriaxone resistance in *E. coli* in this study was lower than that reported in a study conducted in Duhok, Iraq, in which *E. coli* exhibited 100% total resistance to ceftriaxone [22]. The lowest level of bacterial resistance was observed for *Streptococcus *spp., which contradicts the findings of a study conducted in Libya in which the majority of *Streptococcus *spp. were resistant to ceftriaxone [15]. Moreover, this study revealed that the rate of ceftriaxone resistance was significantly associated with the type of bacterial strain. While Gram-negative bacteria had the highest resistance rate than Gram-positive, this might be related to the fact that most of the isolated bacteria in this study were Gram-negative and that there was a statistically significant association between the ceftriaxone resistance rate and the type of isolated bacteria.

Strengths and limitations

This study's strength is that we assessed the ceftriaxone resistance pattern across a four-year period, which gave us a precise representation of the resistance pattern. Additionally, the data was gathered from the PCDR Faculty of Medicine, University of Gezira. This facility serves as the reference laboratory for all Wad Medani hospital settings.

There are some limitations in the current study. In particular, there wasn't a full patient profile available, such as patients' diagnosis, comorbidities and medications administered. Given that the retrospective nature of the study and clinical samples were obtained for diagnostic purposes independently of this study.

## Conclusions

This study reported a high rate of ceftriaxone resistance. The bacteria most resistant to ceftriaxone were *Klebsiella *spp. and *E. coli*. Additionally, resistance to ceftriaxone was significantly correlated with the type of bacterial isolate and the type of bacterial strain. Therefore, hospitals should immediately and significantly modify their antibiotic prescription policy to give doctors a consistent strategy for the rational, safe, and effective administration of antibiotics, and an educational campaign should be strictly implemented to lower the ceftriaxone resistance rate. In addition, antibiotics should be selected depending on the susceptibility pattern of specific microorganisms.

## References

[1] WHO WHO (2024). Global antimicrobial resistance and use surveillance system (‎GLASS)‎ report: 2022. https://www.who.int/publications/i/item/9789240062702.

[2] Mogus Mogus, Alemu Alemu, Bruck H (2015). Antimicrobials use, resistance and containment baseline survey syntheses of findings. https://www.researchgate.net/publication/282822382_Antimicrobials_use_resistance_and_containment_baseline_survey_syntheses_of_findings.

[3] (2024). Antimicrobial resistance. https://www.who.int/news-room/fact-sheets/detail/antimicrobial-resistance.

[4] Nahata MC, Barson WJ (1985). Ceftriaxone: a third-generation cephalosporin. Drug Intell Clin Pharm.

[5] Gelaw LY, Bitew AA, Gashey EM, Ademe MN (2022). Ceftriaxone resistance among patients at GAMBY teaching general hospital. Sci Rep.

[6] Gashe F, Mulisa E, Mekonnen M, Zeleke G (2018). Antimicrobial resistance profile of different clinical isolates against third-generation cephalosporins. J Pharm (Cairo).

[7] Malik MI, Alagab MAM, Maatoug MM (2020). Evaluation of the appropriate use of ceftriaxone in internal medicine wards of Wad Medani teaching hospital in Sudan. Int J Med Sci Clin Invent.

[8] Zaidi AKM, Huskins WC, Thaver D, Bhutta ZA, Abbas Z, Goldmann DA (2020). Hospital-acquired neonatal infections in developing countries. Lancet.

[9] (2024). Antimicrobial resistance: global report on surveillance. https://www.who.int/publications/i/item/9789241564748.

[10] (2024). Antimicrobial resistance in the WHO African Region: a systematic literature review. https://iris.who.int/handle/10665/349223.

[11] Melvin B, James S, April M (2020). M100 Performance Standards for Antimicrobial Sustainability Testing, 30th Edition. https://www.nih.org.pk/wp-content/uploads/2021/02/CLSI-2020.pdf.

[12] Cheesbrough M (2006). District Laboratory Practice in Tropical Countries.

[13] Graham J, Galloway A (2001). ACP best practice no 167. J Clin Pathol.

[14] Kumari S, Bhatia R (2003). Quality Assurance in Bacteriology and Immunology. https://terrance.who.int/mediacentre/data/ebola/training-packages/LQMS/11_cd_rom_quality_assurance_in_bacteriology_and_immunology_2002.pdf.

[15] Nag MSM, Al-Awkally NAM, Abouserwel A, Senossi FM, El-Warred S, Ali MAD (2023). Antimicrobial resistance profile of different clinical isolates against Augmentin, imipenem and ceftriaxone. J Pharm Res Int.

[16] Bushra R, Sial AA, Rizvi M, Shafiq Y, Aslam N, Bano N (2016). Report: sensitivity pattern of ceftriaxone against different clinical isolates. Pak J Pharm Sci.

[17] Ayele AA, Gebresillassie BM, Erku DA, Gebreyohannes EA, Demssie DG, Mersha AG, Tegegn HG (2018). Prospective evaluation of Ceftriaxone use in medical and emergency wards of Gondar university referral hospital, Ethiopia. Pharmacol Res Perspect.

[18] Chanda W, Manyepa M, Chikwanda E (2019). Evaluation of antibiotic susceptibility patterns of pathogens isolated from routine laboratory specimens at Ndola Teaching Hospital: a retrospective study. PLoS One.

[19] Fahim NAE (2021). Prevalence and antimicrobial susceptibility profile of multidrug-resistant bacteria among intensive care units patients at Ain Shams University Hospitals in Egypt-a retrospective study. J Egypt Public Health Assoc.

[20] Azimi T, Maham S, Fallah F, Azimi L, Gholinejad Z (2019). Evaluating the antimicrobial resistance patterns among major bacterial pathogens isolated from clinical specimens taken from patients in Mofid Children's Hospital, Tehran, Iran: 2013-2018. Infect Drug Resist.

[21] Niranjan V, Malini A (2014). Antimicrobial resistance pattern in Escherichia coli causing urinary tract infection among inpatients. Indian J Med Res.

[22] Naqid IA, Balatay AA, Hussein NR, Saeed KA, Ahmed HA, Yousif SH (2020). Antibiotic susceptibility pattern of Escherichia coli isolated from various clinical samples in Duhok city, Kurdistan Region of Iraq. Int J Infect.

